# Strategies for managing left main trunk compression by left atrial appendage clip: a case report

**DOI:** 10.1093/ehjcr/ytad595

**Published:** 2023-11-24

**Authors:** Atsuyuki Mitsuishi, Keisuke Yoshida, Yujiro Miura, Tatsuya Noguchi, Tomoki Furushima

**Affiliations:** Department of Cardiovascular Surgery, Kochi Medical School Hospital, 185-1, Kohasu, Okohcho, Nankoku-shi 783-8505, Kochi Prefecture, Japan; Department of Cardiovascular Surgery, Kochi Medical School Hospital, 185-1, Kohasu, Okohcho, Nankoku-shi 783-8505, Kochi Prefecture, Japan; Department of Cardiovascular Surgery, Kochi Medical School Hospital, 185-1, Kohasu, Okohcho, Nankoku-shi 783-8505, Kochi Prefecture, Japan; Department of Cardiology and Geriatrics, Kochi Medical School Hospital, Nankoku-shi, Japan; Department of Cardiology and Geriatrics, Kochi Medical School Hospital, Nankoku-shi, Japan

**Keywords:** AtriClip, Left atrial appendage, Left main trunk, Stenosis, Compression, Case report

## Abstract

**Background:**

Closure of the left atrial appendage (LAA) using a clip in at-risk patients reduces stroke risk. The rate of LAA closure procedures is increasing worldwide; however, complications have been reported, with coronary compression being one possible lethal complication associated with the anatomical structures around the LAA.

**Case summary:**

A 75-year-old man presented with a diagnosis of a φ50 mm saccular thoracic aortic aneurysm. He had a history of chronic atrial fibrillation and functional tricuspid regurgitation. We performed total arch replacement with an open stent graft, tricuspid ring annuloplasty, left atrium Maze procedure, left atrial plication, and LAA closure using a LAA clip. The blood pressure of the patient dropped after closing the pericardium post-operatively. Coronary artery angiography (CAG) confirmed 90% stenosis at the left coronary main trunk (LMT) origin. Percutaneous coronary intervention (PCI) was performed, and the haemodynamics settled.

**Discussion:**

The distance from the anterior wall of the LAA ostium to the LMT can be a risk for AtriClip-induced LMT compression. A different surgical strategy, such as internal sutures or surgical stapler for LAA closure, should be considered under such a condition. Selecting an appropriately sized AtriClip is essential while using the clip, placing it close to the orifice, and visually checking for compression after insertion to prevent LMT stenosis. When LMT compression by the clip was confirmed, levelling the endocardial adipose tissue with the LAA landing zone, cutting and removing the clip or coronary artery bypass grafting during operation, and PCI during CAG should be considered.

**Learning points**

- When left main trunk (LMT) compression by an AtriClip is confirmed, strategies for management include internal sutures, external sutures, or surgical staplers for left atrial appendage (LAA) closure should be considered.
- When using the AtriClip, select one of an appropriate size, place the clip such that the edge is as close to the orifice as possible, and visually check for compression after insertion to prevent LMT compression.
- When LMT compression by the clip was confirmed, levelling the endocardial adipose tissue with the LAA landing zone, cutting and removing the clip or CABG during operation, and PCI during coronary angiography should be considered.

## Introduction

Usage of AtriClips for left atrial (LA) appendage (LAA) exclusion is increasing worldwide.

In a recent randomized controlled trial of patients undergoing cardiac surgery with a history of atrial fibrillation, combined use of LAA occlusion during surgery and antithrombotic therapy was associated with lower ischaemic stroke or systemic embolic events compared with antithrombic therapy alone.^1^ Coronary complications attributed to AtriClip use have been reported, which are often induced by anatomical complications surrounding the LAA. Preoperative computed tomography (CT) can help assess the risk of coronary compression associated with LAA closure, and several strategies are used for the treatment according to the situation. Herein, we report a case of a patient after open stent total arch replacement, tricuspid ring annuloplasty, and LA maze and LAA clip placement, presenting with left main trunk (LMT) coronary artery compression owing to the LAA treated using percutaneous coronary intervention (PCI).

## Summary figure

**Table:** 

Two hours before vital collapse
We performed total arch replacement with an open stent graft, tricuspid ring annuloplasty with a 28 mm semi-rigid ring, left atrial (LA) maze, LA plication, and LA appendage (LAA) closure using an LAA clip.
Pericardial closure
After the pericardium closure, the patient suddenly exhibited high pulmonary pressure (50/40/35 mmHg), requiring adrenaline administration. We inserted an intra-aortic balloon pump, and the vital signs in the patient temporarily recovered.
Coronary angiography
Coronary complications were suspected, and coronary angiography (CAG) was planned after haemostasis and chest closure. Right coronary artery contrast showed insignificant stenosis. Angiography confirmed 90% stenosis at the origin of the left main trunk (LMT). As the AtriClip was close to the stenosis, it was suspected to have compressed the LMT from outside. We performed an intravascular ultrasound examination of the LMT lesion and confirmed flat stenosis at the LMT origin. A 4.0 × 8 mm drug-eluting stent was placed in the LMT because he had a short LMT (7 mm) with a φ5 mm lumen (according to intravascular ultrasound).
After the surgery
The intra-aortic balloon pump was removed 2 days after surgery. The patient was discharged without a coronary artery event.

## Case presentation

A 75-year-old man presented with a 50 mm saccular thoracic aortic aneurysm without any complaint. He had a history of long-standing persistent atrial fibrillation, functional tricuspid regurgitation, hypertension, and dyslipidaemia. His height, weight, and body mass index were 163 cm, 77 kg, and 29 kg/m^2^, respectively. We performed total arch replacement with an open stent graft (Frozenix, Life Science, Tokyo, Japan), tricuspid ring annuloplasty with a 28 mm semi-rigid ring (Contour 3D, Medtronic, Dublin, Ireland), and LA maze, LA plication, and LAA closure using an LAA clip (AtriClip, AtriCure, West Chester, OH, USA).

**Figure 1:**
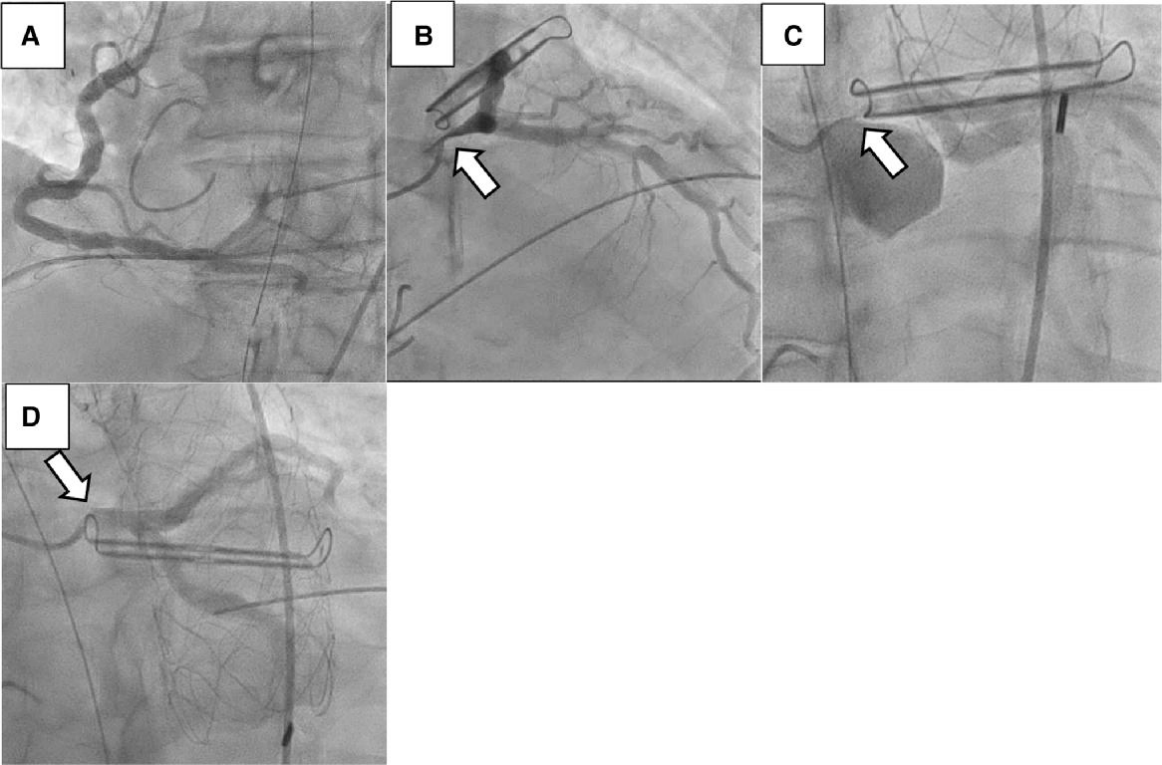
Coronary angiography. (*A*) Right coronary artery and (*B*, *C*, *D*) left main trunk (LMT) with 90% stenosis.

After pericardial closure, he suddenly exhibited high pulmonary pressure (50/40/35 mmHg), requiring adrenaline administration. We inserted an intra-aortic balloon pump (IABP), which temporarily improved his vital signs. If the patient had become haemodynamically unstable after IABP insertion, we would have reopened his chest in the operating room. Diffuse biventricular systolic dysfunction was observed on transoesophageal echocardiography, where the right ventricular wall motion and left ventricular inferior wall motion appeared particularly poor. Coronary complications were suspected, and coronary angiography (CAG) was planned. Right coronary artery contrast showed insignificant stenosis (*Figure 1A*). Angiography confirmed 90% stenosis at the origin of the LMT (*Figure 1B–D*) (see Supplementary material online, *Video S1*). Since the AtriClip was close to the stenosis, it was suspected to have compressed the LMT from outside.

**Figure 2:**
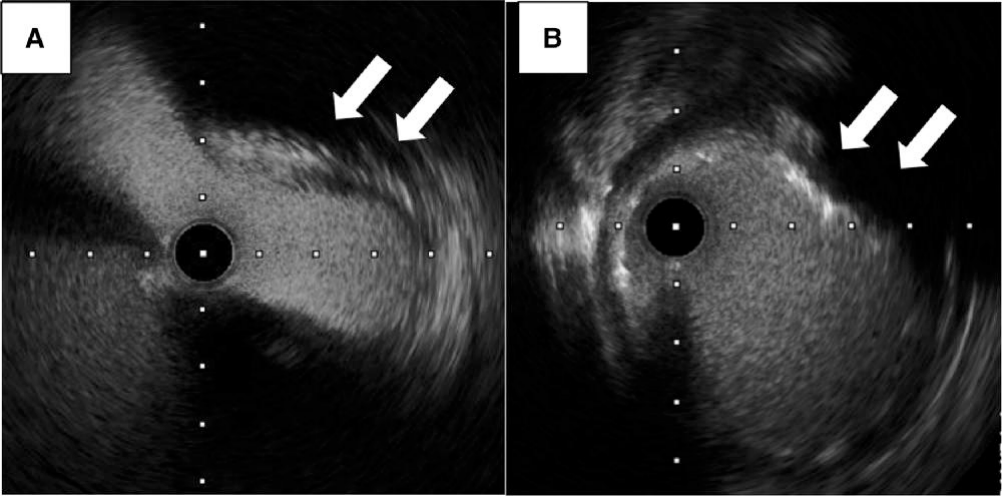
Intravascular ultrasound for examination of the left main trunk compression from outside (white arrows). (*A*) Before percutaneous catheter intervention (PCI) and (*B*) after PCI.

Intravascular ultrasound examination findings of the LMT lesion confirmed flat compression at its origin (*Figure 2*). Thus, dissection at the aortic cannulation site leading to LMT injury was suspected. According to the ultrasound images, compression from external pressure was considered the most likely aetiology. The patient had a short LMT (7 mm) with a φ5 mm lumen. Therefore, a 4.0 × 8 mm drug-eluting stent (XIENCE Skypoint™ Stent, Abbott, Chicago, IL, USA) was placed in the LMT.

**Figure 3:**
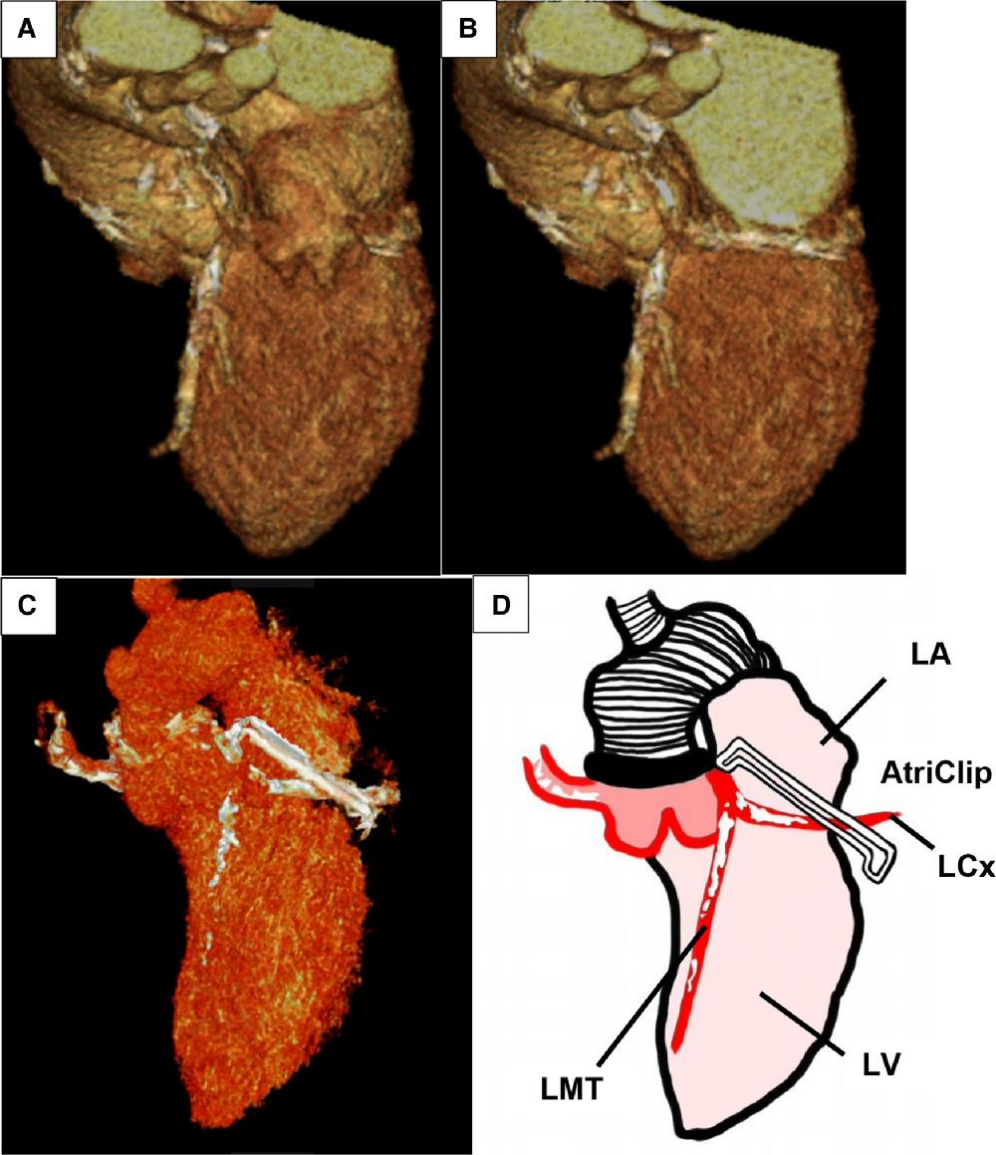
Coronary 3D computed tomography. (*A*) Before surgery with left atrial appendage, (*B*) before surgery without left atrial appendage, (*C*, *D*) after surgery, and (*D*) schematic 3D. LAD, left anterior descending artery; CX, circumflex artery; LA, left atrium; LV, left ventricular.

The patient’s haemodynamics stabilized. After 2 days, the IABP was removed. Post-operative CT showed LMT compression by the AtriClip (*Figure 3*). The patient was successfully discharged. He was followed up for 6 months without any coronary events.

## Discussion

**Figure 4:**
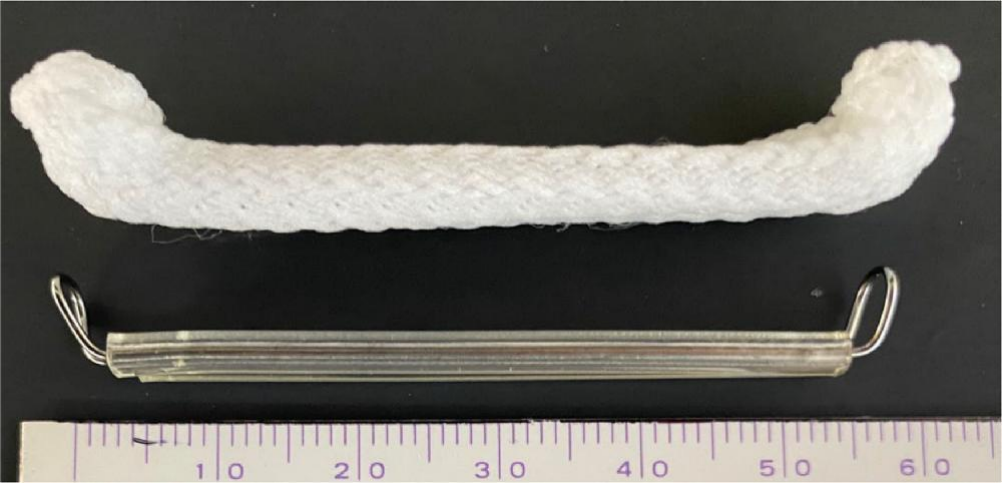
Fifty mm AtriClip that is ∼56 mm long, including the metal parts, and 60 mm with the metal and polyester parts.

Autopsy studies have implicated LAA as the cause of stroke in 90% of non-valvular atrial fibrillation cases.^2^ Several methods, such as internal sutures, external sutures, and surgical staplers for LAA closure, are used for concomitant occlusion during cardiac surgery. The AtriClip comprising two parallel titanium tubes with elastic nitinol springs covered with knit-braided polyester (*Figure 4*) is an option for LAA closure, and coronary occlusion is a potentially fatal complication of this procedure. Contractor *et al.*^3^ reported adverse events associated with the AtriClip. During the timeframe of the study, ∼274 000 AtriClips were sold and nearly 41 000 were being deployed thoracoscopically.^4^ One hundred thirteen adverse events, including four cases of coronary artery damage, three coronary injury–induced deaths, right ventricular dysfunction, and thromboembolism, were attributed to the AtriClips. This may be an underestimation, excluding spontaneously resolving and unreported cases.

Several studies reported treatment strategies for AtriClip-induced coronary compression. For example, Mhanna *et al.*^5^ reported a sudden ventricular fibrillatory arrest due to LMT compression by the AtriClip 2 h after aortic and mitral valve surgery and AtriClip implantation. They performed emergency salvage coronary artery bypass grafting (CABG)×2 with a left internal mammary artery graft to the left anterior descending coronary artery and a saphenous vein graft from the aorta to the second obtuse marginal coronary artery. Kuzmin *et al.*^6^ described the case of patient who underwent mitral and tricuspid valve surgery and AtriClip implantation. The left circumflex artery (LCx) was obstructed at the AtriClip level, even 24 h post-operatively. The compression was treated with PCI through stent implantation. The authors hypothesized that the combined rigid ring and clip may have caused oedema in the tissue between the mitral valve annulus and LAA, causing kinking of the LCx and delayed coronary compression.

Chaldoupi *et al.*^7^ reported two cases of AtriClip-induced coronary compression and suggested less invasive surgery. They resolved LMT and LCx stenosis using thoracoscopy to massage the epicardial fat out of the medial end of the clip and removed the fat to restore the LCx position. They recommended cutting the clip using steel wire cutters or opening both sides of the clip using two firm needle holders if epicardial fat manipulation is insufficient. Thus, knowledge of the device features and preoperative CT can help reduce these complications.

The AtriClip is extended by an additional 3 mm on either side of the device when considering the metal components. The polyester pieces further extend the length by 2 mm. The 50 mm AtriClip was ∼56 mm long, including the metal parts, and 60 mm with the metal and polyester parts (*Figure 4*). This results in the clip protruding 5 mm from the LAA edge at each side. Based on the report^8^ that the mean distance from the anterior wall of the LAA ostium to the LMT is 7.88 ± 2.8 mm, a 50 mm clip selected for a 47 mm LAA will be 3 mm larger than the actual LAA size; therefore, the clip will protrude 8 mm from the LAA edge and compress the LMT. If the distance from the LMT to the LAA orifice is short and/or when selecting a large clip size, the device can compress the LMT. In this case, the distance from the anterior wall of the LAA ostium to the LMT was 7.6 mm, and a 50 mm AtriClip was selected for an LAA slightly shorter than 50 mm. Hence, the clip edge reached the LMT, causing LMT compression.

Furthermore, Mateusz *et al.*^9^ measured the distance from the LAA neck to the LCx surface and considered a distance of <2 mm to indicate a high risk of AtriClip-induced LCx compression. Although the distance between the LAA and LCx differs significantly between men and women, the distance indicating high risk is not sex-dependent.^10^ Their study showed that in 30% of cases, the distance between the LCx and LAA landing zone was <2 mm. Therefore, LAA occlusion should always be carefully performed to avoid iatrogenic complications.

During sternotomy, visually confirming that the clip does not compress the coronary artery is essential to avoid catastrophic haemodynamic collapse. However, we neglected this confirmation.

**Figure 5:**
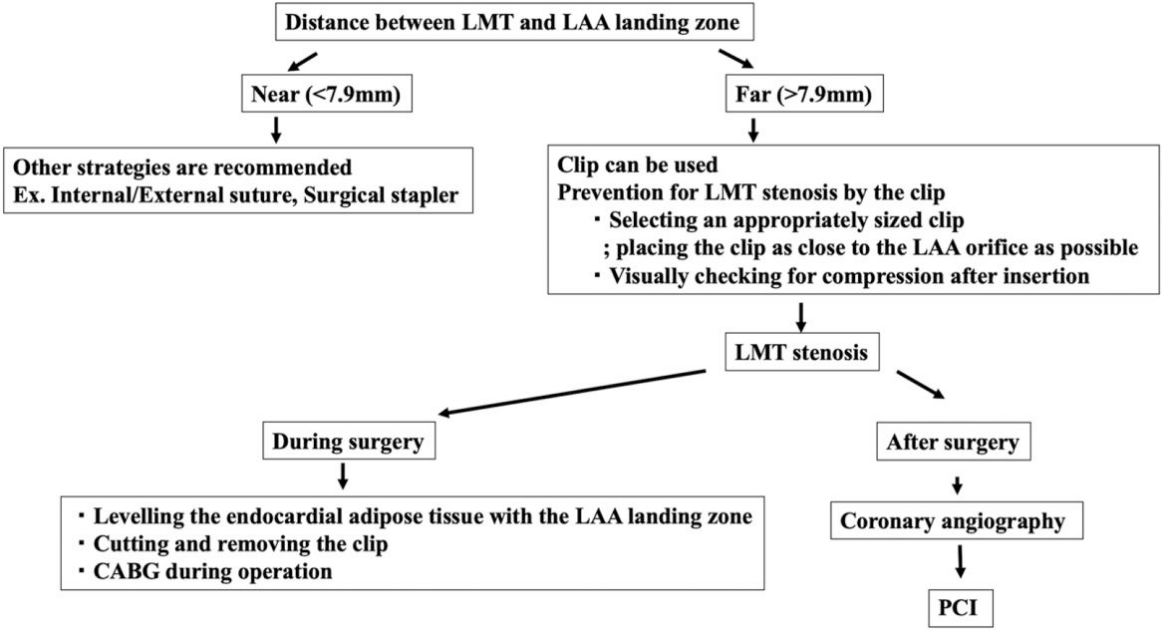
Strategies for managing left main trunk (LMT) compression by left atrial appendage (LAA) clip. CABG, coronary artery grafting; PCI, percutaneous catheter intervention.

The AtriClip is a convenient and effective device; however, a different surgical strategy should be considered in cases where the LMT is near the LAA landing zone. Crucial aspects to prevent LMT compression include selecting an appropriately sized AtriClip, placing it close to the orifice, and visually checking for compression after insertion. When clip-induced LMT compression is confirmed, levelling the endocardial adipose tissue with the LAA landing zone, cutting and removing the clip or CABG during operation, and PCI during CAG should be considered (*Figure 5*). After removal of the clip via opening the clip manually or cutting it, another technique for LAA closure should be used due to the high anatomical risks of LMT compression by a clip.

## Study limitations

First, only one case was included, and the best way to manage AtriClip-induced coronary events remains unclear. We could not determine the definitive anatomical risk factors. Second, perhaps, we should have placed the AtriClip slightly further from the LAA base; however, it could have caused incomplete closures. Third, the long-term outcome is not understood in cases without retrieving the clip compressing the stent. Further research is required to draw more definitive conclusions.

## Conclusion

Coronary compression using the AtriClip should be considered as a differential diagnosis in patients with unpredicted haemodynamic instability after AtriClip implantation, particularly in those with anatomical risks. In such cases, CAG should be performed immediately.

## Supplementary Material

ytad595_Supplementary_Data

## Data Availability

No new data were generated or analysed in support of this research.

## Lead author biography

I’ve graduated from Niigata University, Niigata, Japan. I’m working at Kochi Medical School Hospital, Kochi, Japan. My main area of interest includes cardiovascular surgery.
